# Genome Sequence of *Vibrio cholerae* Strain O1 Ogawa El Tor, Isolated in Mexico, 2013

**DOI:** 10.1128/genomeA.01123-14

**Published:** 2014-10-30

**Authors:** José Alberto Díaz-Quiñonez, Irma Hernández-Monroy, Irma López-Martínez, Joanna Ortiz-Alcántara, Elizabeth González-Durán, Cuitláhuac Ruiz-Matus, Pablo Kuri-Morales, José Ernesto Ramírez-González

**Affiliations:** a Instituto de Diagnóstico y Referencia Epidemiológicos Manuel Martínez Báez, Mexico City, Mexico; b Facultad de Medicina, UNAM, Mexico City, Mexico; c Dirección General de Epidemiología, Mexico City, Mexico; d Subsecretaría de Prevención y Promoción de la Salud, Mexico City, Mexico

## Abstract

We present the draft genome sequence of *Vibrio cholerae* InDRE 3140 recovered in 2013 during a cholera outbreak in Mexico. The genome showed the *Vibrio* 7th pandemic islands VSP1 and VSP2, the pathogenic islands VPI-1 and VPI-2, the integrative and conjugative element SXT/R391 (ICE-SXT), and both prophages CTXφ and RS1φ.

## GENOME ANNOUNCEMENT

Cholera, caused by *Vibrio cholerae*, is a disease characterized by very severe diarrhea and dehydration, which can lead to death in less than 48 hours if left untreated (1). The principal virulence determinant is the potent cholera toxin, encoded by the *ctxAB* genes on the bacteriophage CTXφ found in toxigenic *V. cholerae* genomes. The toxin, together with other virulence factors encoded in clusters of genes called genomic islands, leads to the harmful effects of the *V. cholerae* infection (2).

We report the draft sequence of the genome of *Vibrio cholerae* InDRE 3140 that was collected in Mexico City in August 2013 as an imported cholera case from the Caribbean that later caused an outbreak in the La Huasteca region (3). The strain was isolated from a 46-year-old woman and identified as *V. cholerae* O1 serotype Ogawa, biotype El Tor, on the basis of standard biochemical and serologic testing. Pulsed-field gel electrophoresis and virulence gene amplification (*ctxA*, *ctxB*, *zot*, and *ace*) demonstrated that the strain was different than strains that had circulated in Mexico previously but indistinguishable from the strain that caused outbreaks in Haiti, the Dominican Republic, and Cuba (4, 5).

The *V. cholerae* InDRE 3140 genome was sequenced using the 454 FLX-Titanium platform (Roche, Brandford, CT). A single-end library was generated from genomic DNA and 205,226 reads were obtained. The draft genome was assembled using Newbler version 2.9, with the sequence of *Vibrio cholerae* O1 strain 2010EL-1786 as a mapping reference (CP003069.1 and CP003070.1). Ninety-two contigs were obtained with an *N*_50_ of 118,249 bp and an average coverage of 18.0×. The total sequence length was 4,017,985 bp. The contigs were annotated using the NCBI Prokaryotic Genome Annotation Pipeline with the best-placed reference protein set, GeneMarkS+ method, version 2.6 (revision 440435). The genome showed 3,676 genes, 2,875 coding sequences (CDS), 708 pseudogenes, 25 rRNAs, 67 tRNAs, and 1 noncoding RNA (ncRNA).

Preliminary analyses indicate that *V. cholerae* InDRE 3140 carries the *Vibrio* pathogenic islands VPI-1 and VPI-2, *Vibrio* 7th pandemic islands VSP1 and VSP2, as well as an SXT/R391 (ICE-SXT) integrative and conjugative element, a cholera toxin (CTXφ), and RS1φ prophages. The genomic core is similar to that of the *V. cholerae* O1 El Tor biotype strain 2010 EL-1786, a 2010 Haiti outbreak isolate (6).

A detailed case-control study from cholera case patients as well as the results of comparative and phylogenetic analyses of this genome and other available *V. cholerae* genomes in the La Huasteca region will be published elsewhere.

### Nucleotide sequence accession numbers.

This whole-genome shotgun project has been deposited at DDBJ/EMBL/GenBank under the accession no. JPHJ00000000 and consists of sequences JPHJ01000001 to JPHJ01000092.
